# Biventricular Unloading with Impella and Venoarterial Extracorporeal Membrane Oxygenation in Severe Refractory Cardiogenic Shock: Implications from the Combined Use of the Devices and Prognostic Risk Factors of Survival

**DOI:** 10.3390/jcm10040747

**Published:** 2021-02-13

**Authors:** Georgios Chatzis, Styliani Syntila, Birgit Markus, Holger Ahrens, Nikolaos Patsalis, Ulrich Luesebrink, Dimitar Divchev, Mariana Parahuleva, Hanna Al Eryani, Bernhard Schieffer, Konstantinos Karatolios

**Affiliations:** Department of Cardiology, Angiology and Intensive Care, Philipps University Marburg, 35037 Marburg, Germany; syntila@med.uni-marburg.de (S.S.); birgit.markus79@gmail.com (B.M.); holgerahrens@yahoo.com (H.A.); patsalis@med.uni-marburg.de (N.P.); ulrichluesebrink81@yahoo.com (U.L.); dimitardivchev@yahoo.com (D.D.); mariana.parahuleva@innere.med.uni-giessen.de (M.P.); hanna.aleryani@yahoo.com (H.A.E.); bernschieffer@yahoo.com (B.S.); konstantinoskaratolios@yahoo.com (K.K.)

**Keywords:** impella, VA-ECMO, biventricular unloading, refractory cardiogenic shock, biventricular heart failure, acute heart failure, mechanical support

## Abstract

Since mechanical circulatory support (MCS) devices have become integral component in the therapy of refractory cardiogenic shock (RCS), we identified 67 patients in biventricular support with Impella and venoarterial Extracorporeal Membrane Oxygenation (VA-ECMO) for RCS between February 2013 and December 2019 and evaluated the risk factors of mortality in this setting. Mean age was 61.07 ± 10.7 and 54 (80.6%) patients were male. Main cause of RCS was acute myocardial infarction (AMI) (74.6%), while 44 (65.7%) were resuscitated prior to admission. The mean Simplified Acute Physiology Score II (SAPS II) and Sequential Organ Failure Assessment Score (SOFA) score on admission was 73.54 ± 16.03 and 12.25 ± 2.71, respectively, corresponding to an expected mortality of higher than 80%. Vasopressor doses and lactate levels were significantly decreased within 72 h on biventricular support (*p* < 0.05 for both). Overall, 17 (25.4%) patients were discharged to cardiac rehabilitation and 5 patients (7.5%) were bridged successfully to ventricular assist device implantation, leading to a total of 32.8% survival on hospital discharge. The 6-month survival was 31.3%. Lactate > 6 mmol/L, vasoactive score > 100 and pH < 7.26 on initiation of biventricular support, as well as Charlson comorbity index > 3 and prior resuscitation were independent predictors of survival. In conclusion, biventricular support with Impella and VA-ECMO in patients with RCS is feasible and efficient leading to a better survival than predicted through traditional risk scores, mainly via significant hemodynamic improvement and reduction in lactate levels.

## 1. Introduction

Refractory cardiogenic shock (CS) is a clinical condition of systemic hypotension secondary to cardiac dysfunction with adequate or elevated filling pressures, leading to inadequate perfusion and subsequent failure of end-organs [1]. Prevention or first line treatment of CS includes fluid administration, catecholamines (inotropes and vasopressors) [2]. However, catecholamines may have vasoconstrictive effects on vessels and therefore induce an increase in vascular resistance enhancing the deleterious effects of the CS on the microvasculature, implying that a vasoconstrictor strategy for CS treatment promotes an approach based only on blood pressure, which, while important, does not necessarily reflect cardiac output or tissue perfusion [3,4]. To make things even more complex, current data suggests that CS favors a systemic inflammatory response (SIRS) progressing to multi-organ dysfunction syndrome and subsequent high mortality, so that early recognition of this state with the use of severity scoring systems scores (e.g., Simplified Acute Physiology Score (SAPS II) or Sequential Organ Failure Assessment Score (SOFA)) and aggressive therapy play a crucial role in its prognosis [5]. Despite evolving knowledge and understanding of the pathophysiology, as well as the recent advantages in the pharmaceutical therapy of CS, the mortality of CS remains unacceptably high [2]. The use of mechanical support (MCS) in the setting of acute cardiac failure has emerged as a promising and effective strategy in order to stabilize the heart function without the detrimental effects of catecholamines or to bridge the patients in definite therapies, such as ventricular assist device (VAD) or heart transplantation. The technology available to offer acute hemodynamic support to critically ill patients has evolved exponentially in recent years, whereas the expanding armamentarium of mechanical therapies are becoming increasingly important to provide acute hemodynamic support; Impella and venoarterial Extracorporeal Membrane Oxygenation (VA-ECMO) are the main representatives of this kind of support.

Despite the fact that MCS is becoming increasingly an integral part of the therapy of refractory CS, the best strategy remains unclear. Under the prism of lack of randomized trials, mechanical support has still an IIb recommendation for the therapy according to current guidelines [6,7]. After the results of the Intra-aortic balloon pump (IABP) Shock Trial, IABP should be not utilized any more in this setting, while the use of Tandem Heart due to high invasiveness necessitating a transseptal puncture has been limited [8]. The transvalvular microaxial pump Impella (Abiomed Inc., Danvers, Massachusetts, MA, USA) has emerged as a treatment strategy leading to a reduction in afterload and thus improving cardiac reserve and ventricular metabolism to maintain hemodynamic stability, augmentation of cardiac output, reduction in catecholamine doses, and, subsequently, improvement of perfusion of vital organs [9,10,11,12]. In a recent study, Impella was proved to be superior to standard medical treatment in refractory cardiogenic due to out of hospital cardiac arrest [12]. In the same direction, VA-ECMO is a form of heart-lung bypass machine that offers extended support to patients whose heart or lungs are unable to sustain life in the acute setting, serving as a bridge to myocardial recovery or medical decision, or as a bridge to definitive therapy (VAD or heart transplantation). The VA-ECMO can also be implanted percutaneously and is useful in refractory CS due to uni- or bi-ventricular failure and cardiopulmonary resuscitation (CPR) [13,14]. However, due to the high retrograde flow in the aorta, VA-ECMO may increase left ventricular (LV) afterload leading to an LV extension in patients with severely depressed LV function [15].

In the same direction, the use of MCS devices in patients with severe CS is often related with adverse events and complications, demanding special training and resources [16,17]. The patients with post cardiac arrest CS comprise a group of very ill patients with even worse outcome compared to patients without cardiac arrest [18]. The implementation of predictive models in these patients is a special challenge in the field of intensive care and cardiology. Since no study so far has focused on the prognostic factors of mortality in the particular setting of biventricular mechanical support in patients treated with Impella and VA-ECMO, we aimed to evaluate the independent risk factors of negative outcome in this setting of severely ill patients.

In the current retrospective study, we report our experience from the combined use of Impella (2.5 and CP) and VA-ECMO in patients with severe CS and present the main outcomes of survival and safety in this setting, paying special attention to risk factors of outcome for these patients.

## 2. Materials and Methods

### 2.1. Patients’ Characteristics

**Figure 1:**
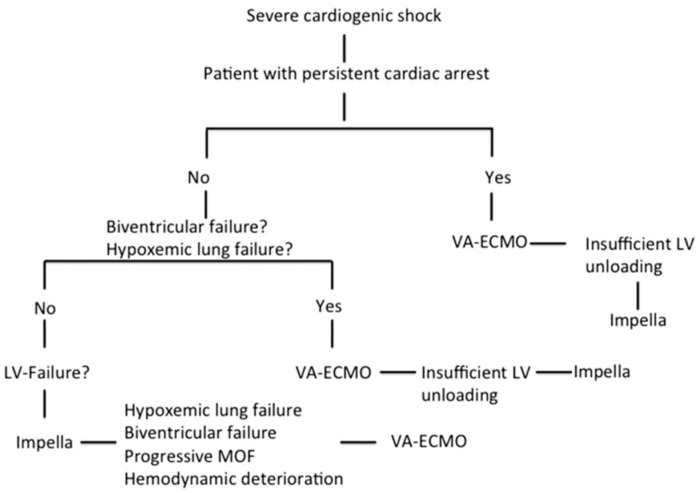
Decision algorithm on mechanical circulatory support in severe cardiogenic shock [19]. (Reproduced with permission from Elsevier).

We retrospectively analyzed data from all patients supported with Impella and VA-ECMO in terms of CS in our institution from February 2013 until December 2019. Among 459 Impella and 267 VA-ECMO patients, we identified 67 patients treated simultaneously with both devices in means of a biventricular approach. The definition of refractory CS was based on persistent hypotension (systolic blood pressure < 90 mmHg or mean arterial pressure < 65 mmHg) despite adequate fluid administration and high doses of inotropes and vasopressors together with sustained evidence of impaired end-organ perfusion and altered mental status. Insufficient LV unloading during VA-ECMO support was defined as worsening pulmonary edema on chest radiography and increasing LV dilation with the presence of spontaneous echo contrast in the LV cavity or insufficient opening amplitude of the aortic valve. RV dysfunction was defined by the dilation of RV and systolic dysfunction. Isolated LV failure was defined by reduced systolic LV ejection fraction without RV dysfunction. The decision algorithm for the choice of MCS in severe CS is based on our institutional algorithm, which was thoroughly described in our previous study (Figure 1) [19].

The study protocol conforms to the ethical guidelines of the 1975 Declaration of Helsinki as reflected in a priori approval by the institution’s human research committee. The local ethics committee waived the need for informed consent due to the retrospective nature of the study. The study adheres to the STROBE guidelines for observational studies.

### 2.2. Implantation of MCS and Management of Patients

All Impella devices were implanted through the femoral artery and placed via the retrograde approach through the aortic valve into the LV under fluoroscopic control in a catheterization laboratory. The VA-ECMO circuit (Maquet Getinge Group), consisting of a centrifugal pump and an membrane oxygenator for complete cardiopulmonary support, was implanted in all cases percutaneously through femoral access in the catheterization laboratory (arterial 17F, venous cannula 21F for women, and 23F for men), since initial observations of VA-ECMO implantation in the intensive care unit (ICU) or in the emergency room was associated with higher prevalence of device associated complications and higher mortality. In all VA-ECMO patients, a distal perfusion cannula was implanted in order to prevent limb ischemia on the side of arterial cannulation for VA-ECMO. All study patients with an acute myocardial infarction (AMI) underwent percutaneous coronary intervention (PCI). The extent of coronary revascularization and adjunctive therapies were left at the operator’s discretion. All out of hospital cardiac arrest (OHCA) patients were treated with targeted temperature management (mild hypothermia of 33–34 °C) for 24 h with an endovascular cooling device (Thermogard XP Temperature Management System, Zoll Medical Corporation, USA). After 24 h, gradual rewarming to 37 °C in hourly increments of 0.25 °C was commenced in all patients. The intention was to maintain a body temperature below or equal to 37 °C until 72 h after cardiac arrest. Inotropes and vasopressors were used to obtain a mean arterial pressure ≥65 mmHg. In all patients level of mechanical support was adjusted to maintain mean arterial pressure ≥65 mmHg with the lowest possible dose of catecholamines and to cover metabolic needs as assessed by central venous oxygen saturation (≥70%) and serum lactate levels (<2.0 mmol/L). A standardized protocol for the management of infections, as well as the kidney function and the indication for renal replacement therapy, was used. Insufficient LV unloading during VA-ECMO was defined as increasing LV distension and impaired aortic valve opening amplitude or severe aortic regurgitation on echocardiographic assessment as well as worsening pulmonary edema. The decision to wean the circulatory support device was based on resolution of shock and clinical assessment. Weaning process was performed by gradually decreasing MCS. Generally, the goal was the weaning of VA-ECMO as first device. Once a low dose of catecholamines with stable mean arterial pressure ≥ 65 mmHg and with low levels of MCS (Impella: Performance level 1 and VA-ECMO: Flow 1.0–1.5 L/min) was achieved, the respective device was removed in ICU and hemostasis was achieved with mechanical compression (St. Jude Medical FemoStop). When weaning of MCS was not possible, bridging to heart transplantation or implantation of VAD was being decided in terms of a heart team approach.

### 2.3. Data Collection and Study End-Points

Intra-hospital clinical data such as time to initiation of MCS devices, catecholamines’ dose, lactate, laboratory parameters, and estimation of ICU severity scores, as well as outcomes and follow-up data were collected from the medical charts. Pre-hospital arrest data were collected with the use of a preformatted standard data collection tool.

Complication rates are also reported. Complications included access site bleeding, limb ischemia and vascular complications requiring surgical or percutaneous repair. Bleeding was defined as blood loss at insertion site requiring blood transfusion. Limb ischemia was defined as clinical hypoperfusion of the leg (decreased skin temperature of the leg or decreased peripheral pulses) requiring treatment or extraction of the device. Vascular complications requiring surgical or percutaneous repair were defined as intervention (surgical or percutaneous) on a vessel dissection, a pseudoaneurysm, an access site thrombosis, or an arteriovenous fistula.

### 2.4. Statistics

All data were analyzed retrospectively. Data are presented as absolute variables and percentages (%) for categorical variables and either median with interquartile range (IQR: 25th–75th percentile) or mean with standard deviation according to the distribution of the variables. We assessed normality using a Shapiro–Wilk test, as well as a Pearson test. After testing for normal distribution, Student’s t-test or Mann–Whitney test was implemented to test for differences between the various characteristics. For categorical variables Fisher’s exact test or chi-square test were used, as appropriate. In order to establish risk factors for mortality on initiation of biventricular support, a univariate analysis was performed to identify the factors that could be associated with the outcome of mortality. All variables from baseline characteristics through hemodynamic parameters on initiation of biventricular support with a *p* value of <0.05 were integrated in a multiple regression analysis model in order to identify the independent risk factors of mortality outcome on initiation of biventricular support. For quantitative parameters, we calculated the cut-off level associated with mortality using receiver operating characteristic (ROC) analysis.

All analyses were made using SPSS 24 (IBM Corp., Armonk, NY, USA) and GraphPad Prism 6.0. (GraphPad Software, San Diego, CA, USA) A two-sided *p*-value < 0.05 was considered statistically significant.

## 3. Results

Our study population consisted of 67 patients. Baseline characteristics of the patients are shown in Table 1. The mean age of the study participants was 61.06 ± 10.7 (years of age) and were predominantly men (80.6%). The main cause of CS was an AMI (74.6%), followed by dilated cardiomyopathy and myocarditis (10.4%). There were 2 cases of severe aortic valve stenosis (2.9%), both of which expired on MCS. The main setting of AMI was ST-elevantion myocardial infarction (STEMI) (72%). The Impella CP was used in one third of the patients (32.8%), while VA-ECMO was the first device that was implanted in most of the cases (58.2%). The majority of patients was resuscitated before hospital admission (65.7%), while 12 patients were admitted under ongoing CPR (17.9% of the total patients). The median time to initiation of first MCS device (t0) was 2 (1–6) hours, the median time to biventricular support (tbiv) was 19.8 (6.7–73.3) hours and time delay from first to second device was 14 (1.3–72) hours. Mean SAPS II score on ICU admission was 73.54 ± 16.03 and increased to 87.54 ± 7.65 prior initiation of biventricular support. Accordingly, SOFA score on ICU admission was 12.25 ± 2.71 and further increased to 13.26 ± 2.65 prior initiation of biventricular support.

**Figure 2:**
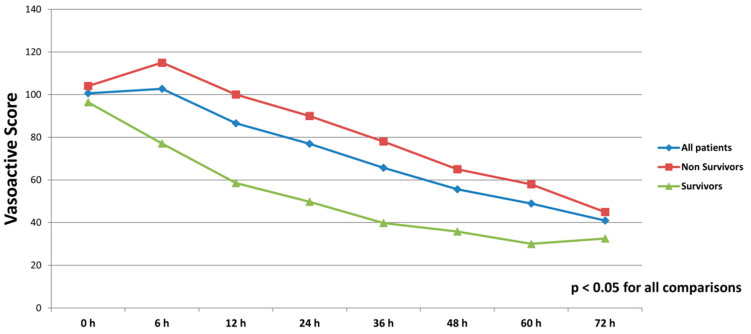
Course of vasoactive score (dobutamine dose (μg/kg/min) + 100 × epinephrine dose (μg/kg/min) + 100 × norepinephrine dose (μg/kg/min)) in all patients, survivors and non-survivors within 72 h after initiation of biventricular support.

**Figure 3:**
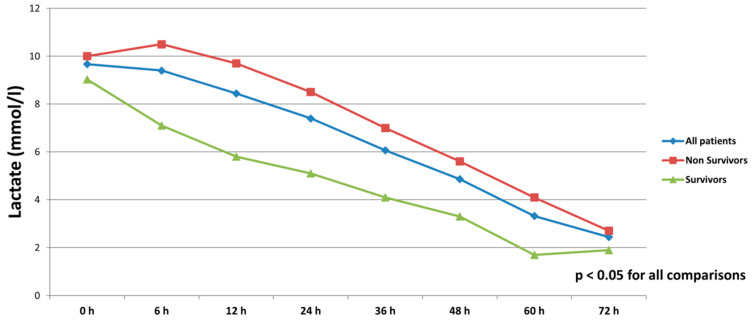
Serum lactate levels in all patients, survivors and non-survivors within 72 h after initiation of biventricular support.

All patients were on catecholamines on admission to ICU (100% norepinephrine, 50.7% dobutamine, and 38.8% on epinephrine) (Table 1). The doses of vasopressors and inotropes increased significantly despite the initiation of the first MCS device (Table 2). After the initiation of the second MCS device, we observed a statistically significant decrease in catecholamines as assessed through the vasoactive score (dobutamine dose (μg/kg/min) + 100 × epinephrine dose (μg/kg/min) + 100 × norepinephrine dose (μg/kg/min)) (Figure 2). The decrease was observed not only among survivors, but also among non-survivors. Serum lactate levels were also significantly reduced within 72 h on biventricular support (Figure 3).

A total of 45 (77.3%) patients expired on ICU, 34 (75.6%) of them had prior CPR. The main causes of death was refractory CS (17 (37.8%) patients), sepsis with multi organ failure (23 (51.1%) patients) and brain death (5 (11.1%) patients)). Overall, 22 (32.8%) patients survived to hospital discharge: 5 (7.5%) patients were successfully transferred to VAD, 17 (25.4%) patients were discharged to cardiac rehabilitation. All latter patients survived to 6 months after ICU discharge, while one VAD patient expired directly after VAD implantation due to an overwhelming sepsis, resulting to 31.3% survival rate on 6 months for the study group.

**Figure 4:**
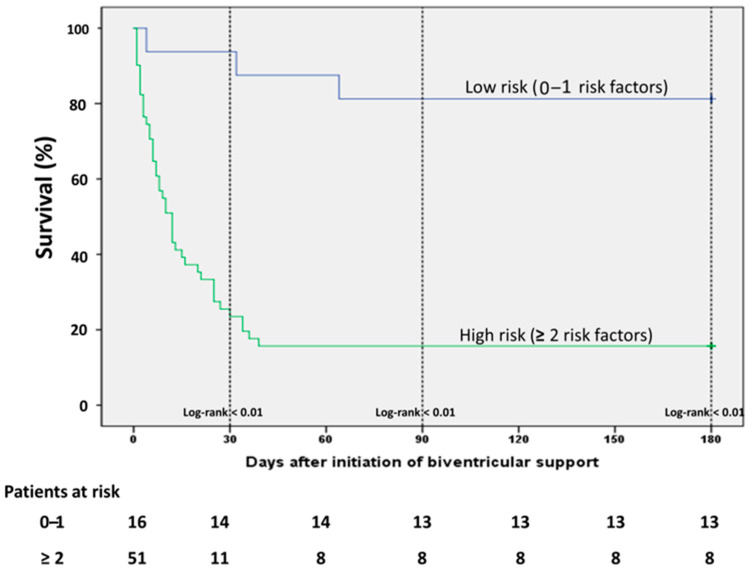
Kaplan Maier Analysis curve among patients in the various risk groups. The patients with none or only one risk factor demonstrate significantly better survival (risk factors: lactate > 6 mmol/L, pH < 7.26, vasoactive score > 100, CCI > 3, prior resuscitation).

On ICU admission, lactate levels were significantly lower and pH significantly higher among survivors, while non-survivors had a higher need of vasopressors as depicted from vasoactive score (Table 1). These differences were consistent even after initiation of the first MCS device and remained statistically significant before initiation of biventricular unloading (Table 2). The non-survivors were significantly older than survivors, whereas an equal distribution was observed among the baseline comorbidities. The age-adjusted Charlson comorbidity Index (CCI) was significantly higher among non-survivors, mainly driven by older age in this group (Table 1). Prior cardiac arrest and VA-ECMO implantation as first device were significantly associated with mortality in the univariate analysis, while the survivors had significantly higher heart rate before escalation with the second MCS device. All factors associated with the outcome of mortality in the univariate analysis were included in a multiple regression analysis model in order to establish risk factors of outcome in these patients. Since we observed that there was a difference in the survival outcomes according to the implantation of the first device, we conducted a simple analysis in order to identify any differences in the baseline characteristics between patients receiving Impella firstly (Impella-first) and patients receiving VA-ECMO firstly (VA-ECMO-first). We observed only few differences between the groups regarding baseline characteristics (Supplementary Table S1). All factors, which differed between Impella-first and VA-ECMO-first and also were associated with mortality (i.e., lactate, prior cardiac arrest), were already incorporated in the multiple regression analysis model. We did not include age in the regression analysis, since the CCI was already age-adjusted. In the multivariate analysis, 5 variables, that is lactate > 6 mmol/L, pH < 7.26, vasoactive score > 100, prior CPR and CCI > 3, were independent risk factors of mortality in this setting. The Horowitz Index presented a statistically marginal association with mortality, however this correlation did not reach a statistical significance of <0.05. All univariate and multivariate correlations are presented on Table 3. In order to evaluate better the impact of these risk factors we divided the patients in two categories (low and high risk) according to the presence of each of these factors and conducted a survival analysis: in patients without or only with one of these factors (low risk) and in patients with 2 or more risk factors. The low risk patients had a statistically significant better survival compared to the patients of the high risk group (Figure 4).

In none of the patients was a major displacement of the device observed. The most frequent observed access site bleeding requiring red blood cells transfusion. Complication requiring intervention or removal of device occurred in 13 patients (19.4%). All survival and complications rates are listed on Table 4.

## 4. Discussion

In this observational study we report our experience and results with the simultaneous use of Impella and VA-ECMO in terms of biventricular MCS in patients with refractory CS To date, very few studies have investigated the combined use of Impella and VA-ECMO in patients with CS, whereas no study so far has concentrated on the identification of multiple risk factors associated with outcome in this particular setting of patients [19,20,21,22].

The main result of our study is that biventricular unloading with Impella and VA-ECMO is feasible and efficient allowing ICU discharge to cardiac rehabilitation or successful bridging to VAD in 22 (32.8%) patients with refractory CS. Moreover, overall survival rates were higher than predicted by mortality risk scores. Our group of patients represents a cohort of critically ill patients. Upon ICU admission, the SOFA score amounted to 12.15 ± 2.71 and the SAPS II score to 73.54 ± 16.03. Despite initial support with the first MCS device, the patients of our study group remained critically ill, as all severity markers, such as ICU mortality scores, vasoactive score, and lactate levels increased significantly. This observation underlines the fact that CS is often a progressive disease with high mortality despite optimal pharmaceutical or initial MCS approach. Prior to initiation of biventricular MCS, the SOFA and SAPS II score increased to 13.26 ± 2.65 and 87.54 ± 7.65, respectively, corresponding to a mortality rate of more than 90%. In our cohort we observed a survival rate of 32.8% on ICU discharge and a long-term survival of 31.3% at 6 months. These survival rates may seem rather low. However, we consider our patients to be at higher risk for death as compared to previous studies with MCS in the setting of CS. In the study of Schrage and colleagues comparing Impella with IABP a survival rate of 51.5% was reported for the Impella group [23]. However, in this study a lower rate of patients were prior resuscitated (35.9 vs. 65.7% in our study); most importantly, an almost one third of our CPR patients were admitted to our institution under ongoing CPR, which consists a major risk factor of adverse outcome. Moreover, our patients presented with higher lactate levels compared to the study of Schrage and colleagues. Similarly, in the study of Alushi and colleagues comparing IABP with Impella in CS with a reported mortality of 52% in the Impella group, the median SAPS II score and the lactate levels were significantly lower compared to our registry [24]. Previous studies on Impella in CS reported mortality rates between 64 and 74% [25,26]. The study by Gaudard and colleagues reported a lower 28-day mortality rate of 43% using Impella in patients with refractory CS [20]. However, in this study an Impella 5.0 was employed, which may supply support of up to 5 lit/min and as such implies a better hemodynamic profile in severe CS compared to Impella 2.5 or CP. On the other side, retrospective analyses and animal models suggest that VA-ECMO in CS improves outcome [27,28,29,30,31,32,33]. In a recent rather small prospective study in CS, VA-ECMO demonstrated a better survival compared to our group [34]. However, the baseline lactate levels are significantly higher in our group, implying a worse hemodynamic and perfusion profile of our patients. Similarly, VA-ECMO demonstrated a better survival rate in patients with CS compared to our study results [13]. However, in this study only 32.1% of the patients were resuscitated prior to hospital admission (compared to 65.7% in our study), while our patients were in high imminent risk of death with higher lactate levels and mortality risk scores. In our investigation we observed a 37.5 % (12 out of 32 patients) survival among CPR patients presenting with return of spontaneous circulation (ROSC), which is higher than the observed survival in previous studies among patients supported with IABP, Impella, or VA-ECMO [25,35]. These data suggests that biventricular unloading with Impella and VA-ECMO may play a role in the management of global myocardial dysfunction after CPR, known as “post-cardiac arrest syndrome’’ [36].

In CS the objectives of MCS are hemodynamic support in order to restore circulation and organ-perfusion, but also to unload the heart allowing myocardial recovery. The latter is not necessarily a goal that can always be fulfilled through VA-ECMO. Due to the retrograde blood flow through the arterial cannula, the arterial system is pressurized and LV afterload increases leading to impaired opening amplitude of the aortic valve. In the setting of depressed LV contractility, the insufficient aortic valve opening increases dramatically wall stress and myocardial oxygen consumption, which in turn are detrimental for myocardial recovery. In our study, 39 patients demonstrated clinical and hemodynamic signs of LV distension, which made the unloading of the LV with an Impella necessary. The management of VA-ECMO induced LV after loading is not standardized and several mechanical approaches (including atrial septostomy, IABP, and Impella) have been suggested to deal with LV distension [15]. A recent meta-analysis demonstrated that LV unloading, irrespective of the used strategy, was associated with decreased mortality in patients with CS supported with VA-ECMO [37]. However, the analysis by Russo and colleagues indicated that unloading with Impella might be superior to IABP [38]. Similarly, in a large recent multicenter study, Impella unloading in patients supported with VA-ECMO was associated with better survival compared to VA-ECMO alone, despite higher rate of vascular complications and higher rate of renal replacement therapy [22]. Finally, randomized trials are needed to define the best unloading strategy. In a recent study, the combined use of Impella with VA-ECMO was superior to VA-ECMO alone in the therapy of CS in a propensity score matching analysis [21], while in previous studies the combined unloading with Impella and VA-ECMO was proved to be safe and feasible [19,39].

The effective biventricular unloading with Impella and VA-ECMO is also strengthened by the significant decrease in vasoactive score and of the lactate levels after initiation of support. Serum lactate levels on admission are well known indicators of hypoperfusion and may offer prognostic information [40,41]. In our study the initiation of the first MCS device failed to restore the hemodynamic and perfusion profile of our patients, since vasopressors and lactate levels were significantly increased. On the other hand, the initiation of biventricular support led to a significant decrease in both vasoactive score and lactate levels, which suggest a reversal of peripheral hypoperfusion and adequate organ-perfusion.

Since data regarding prediction of mortality in patients with biventricular are scarce, we performed a regression analysis in our patients in order to identify independent risk factors of mortality based on the results of the univariate analysis. On initiation of biventricular support serum lactate levels > 6 mmol/L, pH < 7.26, vasoactive score > 100, CCI > 3 and prior CPR were independent predictors of mortality (Table 3). Furthermore, patients with 4–5 risk factors had a significantly worse survival than patients with fewer risk factors (Figure 4). Patients with 0–1 risk factors demonstrated the highest survival rate (Figure 4). Although the prediction of refractory CS to an individual clinical situation will always remain a challenge, the identification of risk factors of outcome might ease the communication of objective prognostic information to family members and surrogate decision makers, help ICU physicians and interventionists to identify severe CS patients with reasonable chance of survival and reduce of futile healthcare. Lactate is a well-known predictor of mortality in cardiogenic shock and serves as an independent predictor in setting of severe CS treated with Impella or VA-ECMO or both [42,43,44], while combined use of lactate and pH demonstrated better prognostic capacity than traditional risk scores in patients treated with VA-ECMO for severe shock [45]. Similarly, vasoactive score served as predictor of mortality in patients treated with Impella 5.0 and biventricular support [20]. In the same direction, data regarding the prognostic role of CCI in shock treated with VA-ECMO are sparse, while no data exist about its role in the prognosis of patients treated with Impella [46]. CS is rather a complex pathophysiological entity characterized by a vicious spiral circle in which ischemia causes myocardial dysfunction, which in turn aggravates the organ hypoperfusion leading to multi-organ insufficiency and death. As such, the identification of single factors of reverse outcome in this setting may not lead to objective prognostication, while such an approach does not necessarily reflect the whole spectrum of the underlying pathophysiology. Under this perspective, the distribution of patients in a model of several risk groups according to the presence of various independent risk factors and the evaluation of the risk in this model consists a major strength of our study.

Data regarding complications of biventricular support with Impella and peripheral VA-ECMO are lacking. In our registry, there was no major device displacement observed. The main complication was access site bleeding requiring transfusion. Moreover, we observed vascular complications requiring percutaneous or surgical repair in 10.4%, whereas in 6 patients (9%) the device had to be removed due to access site adverse events.

### Study Limitations

Our observations are obviously limited by the retrospective and non-randomized design of our study. However, this is the largest single center study so far on biventricular support with Impella and VA-ECMO in refractory CS. In addition, all patients were treated according to our institutional operation procedures and standard algorithms. The 6-month follow-up consists an additional strength of the study. Second, our investigation was a single-center study and our study population was relatively selective with the majority of patients having AMI as cause of the CS and therefore, the results should be interpreted in this context. Nevertheless, coronary artery disease remains the leading cause of the CS. Additionally, detailed hemodynamic data derived from right heart catheterization before and after device implantation were not available for all patients. However, in emergency situations extensive invasive hemodynamic measurements are often not performed. Moreover, we could only retrieve adverse events and complications that were properly documented in the patients’ chart. We, therefore, focused on mortality outcomes as primary end-point that were well documented in our Impella registry. Furthermore, the absence of routine measurement of plasma-free hemoglobin limits the assessment of device-related hemolysis. Lastly, there were not any cases with Impella 5.0. However, this type of Impella needs surgical placement and assumes the existence of cardiothoracic support, meaning that it would be not always feasible in emergency settings or in centers lacking cardiothoracic departments. Given the retrospective nature of this study, the results remain preliminary and all associations need to be evaluated in future prospective and randomized studies.

## 5. Conclusions

In conclusion, biventricular support with Impella and VA-ECMO in patients with refractory CS is feasible and efficient. Biventricular support with VA-ECMO and Impella was associated with improved survival in patients with refractory CS as compared to the predicted mortality assessed by ICU risk scores. Lactate, pH and vasoactive score on initiation of biventricular support, as well as age-adjusted CCI and prior CPR are independent risk factors associated with mortality in this group of patients, while the presence of one or more of these risk factors may offer important prognostic survival information. Future prospective, randomized studies are needed to validate these results and possibly contribute to improvements in the acute management of severe CS.

## Figures and Tables

**Table 1:** Baseline Characteristics of the study participants.

Baseline Characteristics	Total Cohort (*n* = 67)	Survivors (*n* = 22)	Non-Survivors (*n* = 45)	*p*-Value
Age (years)	61.06 ± 10.7	55.68 ± 10.02	63.63 ± 10.14	0.003
Weight (kg)	86.17 ± 13.29	87.64 ± 10.67	85.65 ± 14.68	0.57
BMI (kg/m^2^)	27.64 ± 4	27.59 ± 2.86	27.67 ± 4.47	0.94
Male/female, *n* (%)	54 (80.6)/13 (19.4)	17 (77.3)/5 (22.7)	28 (62.2)/8 (37.8)	0.27
Etiology of cardiogenic shock				
Acute myocardial infarction, *n* (%)	50 (74.6)	17 (77.3)	33 (73.3)	1
STEMI/NSTEMI, *n* (%)	36 (72)/14 (18)	16 (94.1)/1 (5.9)	20 (60.6)/13 (39.4)	0.04
Dilative cardiomyopathy-myocarditis, *n* (%)	7 (10.4)	4 (18.2)	3 (6.7)	0.21
Aortic stenosis, *n* (%)	2 (2.9)	0	2 (4.4)	1
Sepsis/MOF, *n* (%)	5 (7.5)	0	5 (11.4)	0.16
Other (RVOT trauma, postpartum cardiomyopathy, TakoTsubo), *n* (%)	3 (4.4)	1 (4.5)	2 (4.4)	1
Impella first/VA-ECMO first, *n* (%)	28 (41.8)/39 (58.2)	14 (63.6)/8 (36.4)	14 (31.1)/31 (68.9)	0.02
Impella 2.5/CP, *n* (%)	45 (67.2)/22 (32.8)	17 (77.3)/5 (22.7)	28 (62.2)/17 (37.8)	0.18
Prior cardiac arrest, *n* (%)	44 (65.7)	9 (41)	35 (77.8)	0.005
OHCA	26 (59.1)	6 (66.7)	20 (57.1)	
IHCA	18 (13.6))	3 (33.3)	15 (42.9)	
Under CPR on hospital admission	12 (27.3)	2 (22.2)	10 (28.6)	
Prior CAD, *n* (%)	25 (37.3)	6 (27.3)	19 (25.3)	0.28
Prior MI, *n* (%)	15 (22.4)	2 (9.1)	13 (28.9)	0.12
Prior CABG, *n* (%)	5 (7.5)	2 (9.1)	3 (6.5)	1
Prior Hypertension, *n* (%)	51 (76.1)	18 (81.8)	33 (73.3)	0.55
Prior Diabetes, *n* (%)	21 (31.3)	4 (18.2)	17 (37.8)	0.16
Prior COPD, *n* (%)	5 (7.5)	2 (9.1)	3 (6.5)	1
Prior Stroke, *n* (%)	12 (17.9)	6 (27.3)	6 (13)	0.19
Prior PAD, *n* (%)	6 (9)	0 (0)	6 (13)	0.17
CCI	2 (3–5)	2 (1–4)	3 (2–5)	0.02
ICU stay (days)	14.5 (5–28.75)	26.27 ± 14.44	9.5 (4–24)	0.001
Hemodynamic variables on ICU admission				
Heart rate (bpm)	93.68 ± 27.31	90.9 ± 29.1	95 ± 26.7	0.56
Systolic blood pressure (mmHg)	88.49 ± 26.6	95 ± 25.54	85.37 ± 26.8	0.16
Diastolic blood pressure (mmHg)	55.09 ± 14.93	55.91 ± 14.28	54.7 ± 15.36	0.76
Noradrenaline				
*n* (%)	67 (100)	22 (100)	45 (100)	1
(μg/kg/min)	0.5 (0.14–0.79)	0.33 (0.2–1.3)	0.5 (0.2–0.77)	0.21
Dobutamine				
*n* (%)	34 (50.7)	12 (64.5)	22 (48.9)	0.8
(μg/kg/min) *	5.79 ± 2.44	5.35 ± 1.1	6 ± 2.9	0.45
Epinephrine				
*n* (%)	26 (38.8)	10	16	0.59
(μg/kg/min) *	0.4 (0.21–3.8)	0.17 ± 0.1	0.33 (0.1–0.48)	0.1
Vasoactive score (μg/kg/min)	59 (19–117)	69.6 (32.7–91.6)	105.2 (60.5–157.5)	0.005
Blood values on ICU admission				
pH	7.36 ± 0.16	7.38 ± 0.12	7.24 ± 0.18	0.002
Lactate (mmol/L)	8.8 ± 6.7	5.88 ± 5.33	11.03 ± 7.99	0.008
Creatinine (mg/dL)	1.78 ± 0.8	1.77 ± 0.44	1.78 ± 0.94	0.95
GFR (ml/min)	43.6 ± 15.7	44.1 ± 15.2	43.14 ± 15.9	0.81
Bilirubin (mg/dL)	1.2 (0.73–1.6)	0.9 (0.6–2.9)	1.2 (0.78–1.6)	0.6
Clinical variables o ICU admission				
LVEF (%)	35.4 ± 3.9	34.4 ± 3.6	36.02 ± 3.9	0.12
Horowitz index	222 (161–461)	312 (192–462)	211.7 (156–429)	0.17
SOFA	12.25 ± 2.71	11.95 ± 3	12.39 ± 2.58	0.54
SAPS II	73.54 ± 16.03	71.55 ± 12.64	74.5 ± 17.46	0.48
Time to implantation of first device (t0) (h)	2 (1–6)	2 (0.97–3.5)	2 (0.92–7.44)	0.61
Time to biventricular support (tbiv) (h)	19.8 (6.7–73.3)	15 (7.5–65)	22 (4–91)	0.36
Time from first MCS device to biventricular support (t0-biv) (h)	14 (1.3–72)	11.5 (1.7–88)	18 (0.6–68)	0.7
Duration of biventricular support (h)	96 (24–186)	96 (24–168)	72 (24–192)	0.97

BMI: body mass index; STEMI: ST-elevation myocardial infarction; MOF: multi-organ failure, RVOT: right outflow ventricular tract; VA-ECMO: venoarterial Extracorporeal Membrane Oxygenation; OHCA: out of hospital cardiac arrest, IHCA: in hospital cardiac arrest; CPR: cardiopulmonary resuscitation; CAD: coronary artery disease; MI: myocardial infarction; CABG: coronary artery bypass graft; COPD: chronic obstructive pulmonary disease; PAD: peripheral artery disease; CCI: Charlson Comorbidity Index, age-adjusted; ICU: intensive care unit; GFR: glomerular filtration rate; GFR: Glomerular filtration rate; LVEF: left ventricular ejection fraction; SOFA: sequential organ failure assessment; SAPS II: simplified acute physiology score II; MCS: mechanical circulatory support. Numbers are presented as mean (± standard deviation) or median (interquartile range. IQR 25th–75th percentile) or frequency (percentile). * doses refer to patients receiving the index medicament.

**Table 2:** Variables prior initiation of biventricular support.

Hemodynamic Variables	Total Cohort (*n* = 67)	Survivors (*n* = 22)	Non-Survivors (*n* = 45)	*p*-Value
Heart rate (bpm)	107.2 ± 28.59	117.2 ± 24.40	102.9 ± 29.45	0.046
Systolic blood pressure (mmHg)	86.63 ± 19.67	86.18 ± 13.71	86.85 ± 22.09	0.9
Diastolic blood pressure (mmHg)	55.5 ± 14	59.27 ± 12.06	53.70 ± 14.62	0.13
Mean blood pressure (mmHg)	65.88 ± 14.08	68.24 ± 11.28	64.75 ± 15.22	0.34
Noradrenaline				
*n* (%) (μg/kg/min)	67 (100) 0.73 ± 0.48	22 (100) 57.90 ± 32.17	45 (100) 66.89 ± 37.66	1 0.36
Dobutamine *				
*n* (%) (μg/kg/min) *	54 (80.6) 6.76 ± 3.2	16 (72.7) 5.97 (3.7–7.87)	38 (84.4) 7 (5.15–8.13)	0.21
Epinephrine				
*n* (%) (μg/kg/min) *	40 (59.7) 0.2 (0.12–0.5)	15 (6.8) 0.15 (0.07–0.43)	25 (55.6) 0.2 (0.15–0.54)	0.21
Vasoactive score (μg/kg/min)	89 (53.99–154)	69.62 (32.7–91.57)	105.2 (60.3–157.5)	0.02
Blood values				
pH	7.29 ± 0.17	7.38 ± 0.12	7.24 ± 0.18	0.001
Lactate (mmol/L)	9.67 ± 7	4.8 (2.9–5.6)	9 (4.4–16.2)	0.005
Creatinine (mg/dL)	1.85 ± 0.65	1.86 ± 0.72	1.84 ± 0.63	0.92
GFR (mL/min)	39.4 ± 17.09	40.36 ± 16.68	38.93 ± 17.45	0.75
HCO^3−^ (mmol/L)	18.16 ± 6.2	20.38 ± 5.9	17.1 ± 6.13	0.05
Bilirubin (mg/dL)	1.11 (0.7–1.9)	1.11 (0.4–1.61)	1.2 (0.92–1.9)	0.48
Clinical variables				
LVEF (%)	28.5 ± 4.3	28.24 ± 3.32	28.67 ± 4.1	0.67
Horowitz index	208 (85.5–276)	254 (112.1–377.8)	161 (62.5–236.4)	0.03
SOFA	13.26 ± 2.65	13.68 ± 2.73	13.07 ± 2.61	0.37
SAPS II	87.54 ± 7.65	88.18 ± 8.55	87.24 ± 7.26	0.64

GFR: Glomerular filtration rate; LVEF: left ventricular ejection fraction; SOFA: sequential organ failure assessment; SAPS II: simplified acute physiology score II. Numbers are presented as mean (± standard deviation) or median (interquartile range. IQR 25th–75th percentile) or frequency (percentile). * refer to patients receiving the index medicament.

**Table 3:** Univariate and multivariate analysis of risk factors associated with mortality.

Variable	Univariate Analysis		Multivariate Analysis	
	Odds Ratio (5–95% C.I.)	*p*-Value	Odds Ratio (5–95% C.I.)	*p*-Value
CCI > 3	4.29 (1.44–12.76)	0.009	1.8 (1.07–3.04)	0.03
VA-EMO-first	6.78 (2.1–21.7)	0.001	0.88 (0.28–2.78)	0.8
Prior CPR	7 (2.413–20.31)	0.001	6.5 (1.3–33.3)	0.03
pH < 7.26	9.5 (2.45–36.87)	0.0005	4.67 (1.04–32.3)	0.04
Lactate > 6 (mmol/L)	8.37 (2.55–27.45)	0.0002	11 (1.3–94.7)	0.03
Heart rate > 100 (bpm)	0.3 (0.09–0.91)	0.04	0.32 (0.1–1.03)	0.07
Vasoactive score > 100	6.16 (1.91–19.84)	0.002	4.6 (1.02–28.95)	0.047
HCO ^3−^ < 19 (mmol/L)	2.88 (1.01–8.3)	0.05	0.7 (0.2–2.3)	0.55
Horowitz index	3.21 (1.1–9.44)	0.04	4.4 (0.95–32.29)	0.056

C.I.: confidence interval; CCI: Charlson Comorbidity Index, age-adjusted; VA-ECMO-first: venoarterial Extracorporeal Membrane Oxygenation; CPR: cardiopulmonary resuscitation. Numbers are presented as Odds ratio with confidence intervals (5th–95th).

**Table 4:** Survival and safety outcomes.

Survival at hospital discharge, *n* (%)	22 (32.8)
Survival at 6-months, *n* (%)	21 (31.3)
Bridged to VAD, *n* (%)	5 (7.4)
Major device malfunction, *n* (%)	0 (0)
Access site bleeding needing transfusion, *n* (%)	35 (52.2)
RBC transfusion on MCS (units per day)	1.13 (0.68–1.88)
Myocardial infarction, *n* (%)	0 (0)
Stroke, *n* (%)	0 (0)
Pericardial effusion requiring paracentesis, *n* (%)	1 (1.5)
Limb ischemia or bleeding needing operation or intervention, *n* (%)	7 (10.4)
Limb ischemia or bleeding needing device removal, *n* (%)	6 (9)

VAD: ventricular assist device; RBC: red blood cell; MCS: mechanical support.

## Data Availability

The data presented in this study are available on request from the corresponding author. The data are not publicly available due to ethical restrictions.

## Supplementary Materials

The following are available online at https://www.mdpi.com/2077-0383/10/4/747/s1, Table S1: Baseline characteristics of the study participants according to device implanted firstly.
