# Supplementary material for: Biventricular Unloading with Impella and Venoarterial Extracorporeal Membrane Oxygenation in Severe Refractory Cardiogenic Shock: Implications from the Combined Use of the Devices and Prognostic Risk Factors of Survival
Source: J Clin Med. 2021 Feb 13;10(4):747. doi: 10.3390/jcm10040747 (PMC7918629; doi:10.3390/jcm10040747)
Supplement: Supplementary file 1 [file jcm-10-00747-s001.pdf]

Supplementary Table 1. Baseline Characteristics of the study participants according to device implanted firstly

**Baseline Characteristics**	**Total cohort (N=67)**	**Impella-first (N=28)**	**VA-ECMO-first (N=39)**	**p-value**
Age (years)	61.06 ± 10.7	65.86 ± 8.94	57.69 ± 10.78	0.002
Weight (kg)	86.17 ± 13.29	85.82 ± 15.60	86.63 ± 11.94	0.81
BMI (kg/m^2)	27.64 ± 4	27.52 ± 4.46	27.73 ± 3.71	0.84
Male/Female, n (%)	54 (80.6) / 13 (19.4)	23 (82.1) / 5 (17.9)	31 (79.5) / 8 (21.5)	1
Etiology of cardiogenic shock				
- acute myocardial Infarction, n (%)	50 (74.6)	23 (82.1)	27 (69.2)	0.27
-- *STEMI/NSTEMI, n (%)*	*36 (72) / 14 (18)*	17 (73.9) / 6 (26.1)	19 (70.4) / 8 (29.6)	1
- dilative cardiomyopathy-myocarditis, n (%)	7 (10.4)	3 (10.7)	4 (10.3)	1
- aortic stenosis, n (%)	2 (2.9)	0 (0)	2 (5.1)	0.51
- sepsis/MOF, n (%)	5 (7.5)	2 (7.1)	3 (7.7)	1
- other (RVOT Trauma, postpartum cardiomyopathy, TakoTsubo), n (%)	3 (4.4)	1 (3.6)	2 (5.1)	1
Impella 2.5/CP, n (%)	45 (67.2) / 22 (32.8)	12 (48.6) /16 (51.4)	33 (84.6) / 6 (15.4)	0.0005
Prior cardiac arrest, n (%)	44 (65.7)	16 (57.1)	28 (71.8)	0.048
- *OHCA*	*26 (59.1)*	*9 (56.3)*	*17 (60.7)*	*1*
- *IHCA*	*18 (13.6))*	*7 (43.7)*	*11 (49.3)*	
- *under CPR on hospital admission*	*12 (27.3)*	*0 (0)*	*12 (43.3)*	*0.016*
Prior CAD, n (%)	25 (37.3)	10 (35.7)	15 (38.5)	1
Prior MI, n (%)	15 (22.4)	5 (17.9)	10 (25.6)	0.56
Prior CABG, n (%)	5 (7.5)	1 (3.6)	4 (10.3)	0.39
Prior Hypertension, n (%)	51 (76.1)	22 (78.6)	29 (74.3)	0.78
Prior Diabetes, n (%)	21 (31.3)	7 (25)	14 (35.9)	0.43
Prior COPD, n (%)	5 (7.5)	1 (3.6)	4 (10.3)	0.39
Prior Stroke, n (%)	12 (17.9)	4 (14.3)	8 (20.6)	0.75
Prior PAD, n (%)	6 (9)	1 (3.6)	5 (12.8)	0.39
ICU stay (days)	14.5 [5 – 28.75]	18.71 ± 13.59	17.67 ± 16.98	0.79

**Hemodynamic variables on ICU admission**				
Heart rate (bpm)	93.68 ± 27.31	92.86 ± 26.79	94.10 ± 28.35	0.86
Systolic Blood pressure (mmHg)	88.49 ± 26.6	88.89 ± 22.93	87.78 ± 30.13	0.87
Diastolic Blood Pressure (mmHg)	55.09 ± 14.93	57.41 ± 14.24	53.47 ± 16.16	0.32
Noradrenaline				
n (%)	*67 (100)*	28 (100)	39 (100)	1
(µg/kg/min)	*0.5 [0.14 – 0.79]*	0.21 [0.12 – 0.71]	0.5 [0.2 – 1.17]	0.17
Dobutamine				
n (%)	*34 (50.7)*	16	18	0.06
*(µg/kg/min)**	*5.79 ± 2.44*	6.11 ± 2.87	5.5 ± 2.04	0.48
Epinephrine				
n (%)	*26 (38.8)*	9	17	1
*(µg/kg/min)**	*0.4 [0.21 – 3.8]*	0.31 [0.14 – 0.38]	0.12 [0.08 – 0.34]	0.27
Vasoactive Score (µg/kg/min)	59 [19 - 117]	42 [13 - 83]	57 [38 - 124]	0.26
**Blood values on ICU admission**				
pH	7.36 ± 0.16	7.39 ± 0.19	7.34 ± 0.13	0.21
Lactate (mmol/l)	8.8 ± 6.7	4.55 [2.8 – 8.75]	9.2 [5.9 – 14.9]	0.006
Creatinine (mg/dl)	1.78 ± 0.8	1.71 [1.27 – 1.98]	1.73 [1.39 – 1.96]	0.58
GFR (ml/min)	43.6 ± 15.7	42.5 ± 18.05	43.73 ± 13.70	0.75
Bilirubin (mg/dl)	1.2 [0.73 – 1.6]	1.32 [0.61 – 1.65]	1.11 [0.73 – 1.6]	0.69
**Clinical Variables o ICU admission**				
LVEF (%)	35.4 ± 3.9	32.7 ± 3.62	37.41 ± 2.55	<0.001
Horowitz Index	222 [161 - 461]	232 [176.2 - 515]	212 [160.2 – 446.5]	0.16
SOFA	12.25 ± 2.71	12.32 ± 2.53	12.2 ± 2.86	0.86
SAPS II	73.54 ± 16.03	74.82 ± 16.12	72.65 ± 16.11	0.59
Time to implantation of first device (t0) (hours)	2 [1 - 6]	2.55 [0.95 – 3.5]	2 [0.97 - 11]	0.44
Time to biventricular support (tbiv) (hours)	19.8 [6.7 – 73.3]	18.6 [7 - 69]	21 [6.3 - 79]	0.8
Time from first MCS device to biventricular support	14 [1.3 - 72]	13 [0.9 - 78]	14.8 [1.7 - 69]	0.9

(t0-biv) (hours)				
Duration of biventricular support (hours)	96 [24 - 186]	72 [24 - 144]	96 [24 - 192]	0.32

BMI: body mass index; PAD: peripheral artery disease; COPD: chronic obstructive pulmonary disease; GFR: glomerular filtration rate; PCI: percutaneous coronary intervention; CABG: coronary artery bypass graft; CPR: cardiopulmonary resuscitation; LVEF: left ventricular ejection fraction; GFR: Glomerular filtration rate; STEMI: ST-elevation myocardial infarction; ICU: intensive care unit; SAPS II: simplified acute physiology score II; SOFA: sequential organ failure assessment; MOF: multi-organ failure, RVOT: right outflow ventricular tract; OHCA: out of hospital cardiac arrest, IHCA: in hospital cardiac arrest; CAD: coronary artery disease; CCI: Charlson Comorbidity Index, age-adjusted. Numbers are presented as mean (± standard deviation) or median [interquartile range. IQR 25th – 75th percentile] or frequency (percentile)

*doses refer to patients receiving the index medicament
